# Neuregulin 1 improves cognitive deficits and neuropathology in an Alzheimer’s disease model

**DOI:** 10.1038/srep31692

**Published:** 2016-08-25

**Authors:** Jiqing Xu, Fred de Winter, Catherine Farrokhi, Edward Rockenstein, Michael Mante, Anthony Adame, Jonathan Cook, Xin Jin, Eliezer Masliah, Kuo-Fen Lee

**Affiliations:** 1 Clayton Foundation for Peptide Biology Laboratories, The Salk Institute, La Jolla, CA 92037, USA; 2 Department of Neurosciences, University of California at San Diego, La Jolla, CA 92093, USA; 3 Molecular Neurobiology Laboratories, The Salk Institute, La Jolla, CA 92037, USA

## Abstract

Several lines of evidence suggest that neuregulin 1 (NRG1) signaling may influence cognitive function and neuropathology in Alzheimer’s disease (AD). To test this possibility, full-length type I or type III NRG1 was overexpressed via lentiviral vectors in the hippocampus of line 41 AD mouse. Both type I and type III NRG1 improves deficits in the Morris water-maze behavioral task. Neuropathology was also significantly ameliorated. Decreased expression of the neuronal marker MAP2 and synaptic markers PSD95 and synaptophysin in AD mice was significantly reversed. Levels of Aβ peptides and plaques were markedly reduced. Furthermore, we showed that soluble ectodomains of both type I and type III NRG1 significantly increased expression of Aβ-degrading enzyme neprilysin (NEP) in primary neuronal cultures. Consistent with this finding, immunoreactivity of NEP was increased in the hippocampus of AD mice. These results suggest that NRG1 provides beneficial effects in candidate neuropathologic substrates of AD and, therefore, is a potential target for the treatment of AD.

Alzheimer’s Disease (AD) is characterized by the degeneration of neurons in the hippocampus and cortex, and the appearance of neuritic plaques and neurofibrillary tangles^1^,^2^,^3^. Although the precise cause of AD remains unclear, and is in fact most likely from multiple etiologies. Aggregated Aβ-peptides, resulting from proteolytic cleavage of the amyloid precursor protein (APP), constitute a prime neurotoxic component of senile plaques in the brains of AD patients. Several therapeutic approaches are aimed at reducing Aβ load and neutralizing Aβ toxicity, including passive immunization with Aβ^4^,^5^, preventing aggregation of Aβ^6^, inhibiting Aβ production using β- and γ-secretase inhibitors or siRNA^7^, increasing levels of Aβ-degrading enzymes such as Neprilysin (NEP)^8^, insulin-degrading enzyme^9^ or cathepsin^10^ and augmenting anti-oxidation capacity. Over the past several years, a consensus has emerged that a cocktail of drugs influencing multiple mechanisms may be required to effectively treat AD.

Through alternative splicing of the neuregulin 1 (NRG1) primary mRNA transcript, several subtypes are produced as transmembrane (TM) precursor proteins^11^. Type I (also called neu differentiation factor and acetylcholine receptor-inducing activity) and type II (glial growth factor) NRG1 isoforms contain an Ig domain and an epidermal growth factor (EGF)-like domain, but differ by the presence of a Kringle domain in type II NRG1. Proteolytic cleavage in the extracellular domain near the TM domain of type I and type II NRG1s yields soluble ligands that activate ErbB receptors. Type III (sensory and motor neuron-derived factor) NRG1 isoforms contain an EGF-like domain and a unique cysteine-rich domain that is postulated to serve as a secondary TM domain. Recent evidence suggests that dual cleavage of type III NRG1 by BACE1 and ADAM17 liberates its EGF-like domain and permits paracrine signaling^12^. NRG1 and its cognate receptor ErbB2/ErbB3 and ErbB2/ErbB4 heterodimers or ErbB4 homodimers mediate diverse signaling pathways in neural development and function^13^.

Several lines of evidence suggest that NRG1 itself, or manipulation of NRG1 signaling, may influence cognitive function and neuropathology in AD. First, a single nucleotide polymorphism (SNP) of the NRG1 gene (rs392499) previously found in schizophrenia families is associated with late onset AD with psychosis in U.S. patients^14^. Interestingly, NRG3, another member of the NRG family^15^, is associated with the risk and age at onset of AD^16^. Second, expression of erbB1-4 is altered in mouse models of AD^17^,^18^,^19^. Third, type I NRG1 down-regulates and increases turnover of APP in C2C12 cells^20^. Fourth, NRG1 is neuroprotective against focal cerebral ischemia^21^ and prevents PC12 cell death induced by Aβ^22^. Finally, Aβ reduces spine density^23^, whereas NRG1 signaling maintains spine morphology and density^24^. Collectively, these data suggest that NRG1 signaling may influence Aβ load, synaptic integrity, neuroprotection and cognitive function in AD.

In the present study, we showed that overexpression of either type I or type III NRG1 improves cognitive deficits and ameliorates neuropathology in AD mice^25^. Furthermore, we showed that NRG 1 significantly increases the expression of NEP in neuronal cultures. These results suggest that NRG1 is a potential target for the treatment of AD.

## Results

### NRG1 improves deficits in Morris water maze behavioral test

**Figure 1:**
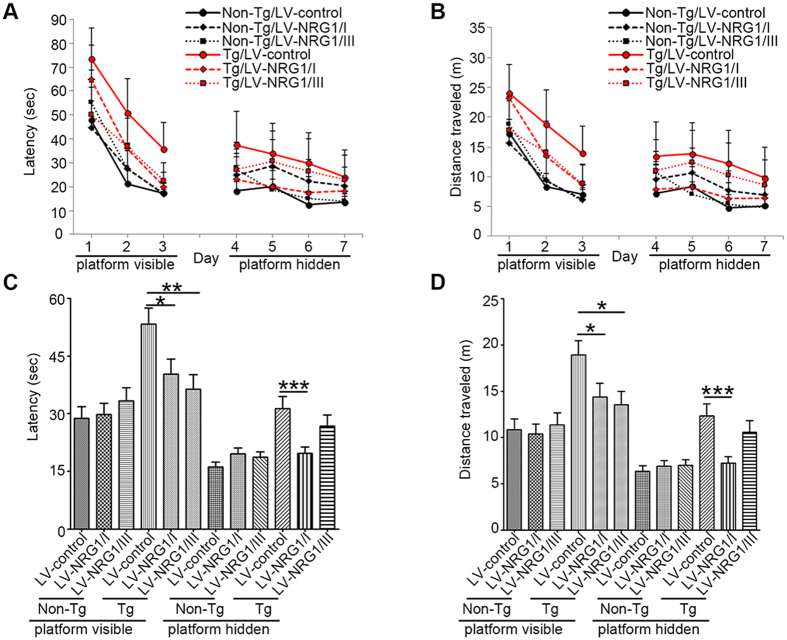
NRG1 improves deficits in Morris water maze behavioral task in APP mice. Eight weeks after stereotaxic injection of female LV-Control, LV-NRG1/I or LV-NRG1/III mice, cognitive function was assessed by the Morris water maze (N = 6). Mice were trained on the visible platform and then tested for spatial learning. Linear regression analysis shows the slope for the latency (**A**) and distance (**B**). Latency or distance across all four trials daily from days 1–3 for visible platform and from days 4–7 for hidden platform was calcuated and expressed as mean ± SEM. A main effect for treatment was observed for latency (**C**) and distance traveled (**D**). *p < 0.05, **p < 0.01, ***p < 0.0005.

To test whether exogenous NRG1 improves congnitive function in AD post-symptomatically, control lentiviruses (LV-control) and lentiviruses expressing full-length rat β1α NRG1 type I (LV-NRG1/I) or type III (LV-NRG1/III) under the CMV promoter were generated and stereotaxically injected into the hippocampus of 7-month old female line 41 transgenic mice expressing a mutated human APP (APP-Tg)^25^ and female non-TG control littermates. Eight weeks after viral injection, performance in the Morris water maze was evaluated. Latency to find the escape platform (Fig. 1A) and distance traveled (Fig. 1B) during the visible and hidden platform phases were measured. The means of latency or distance across all four trials daily from days 1–3 for visible platform and from days 4–7 for hidden platform were calcuated^26^. Differences among means of the six groups were assessed by repeated measures ANOVA followed by Fisher’s least significant difference post-hoc test with level of significance set at p < 0.05. Post-hoc analyses revealed that APP-Tg controls were significantly delayed in finding the escape platform compared to the APP-Tg group injected with type I or type III NRG1 and non-Tg littermates injected with LV-control during the visible platform phase of learning. During the hidden platform phase, the APP-Tg group injected with LV-NRG1/I showed a statistically significant improvement in latency and distance compared to the APP-Tg group injected with the LV-control (Fig. 1C,D). The APP-Tg group injected with NRG1 type III showed a trend of improvement compared with the APP-Tg controls. Distance traveled was also higher in the APP-Tg controls compared to all other groups, consistent with an increase in latency with actively searching for the escape platform. These results support that both type I and type III NRG1s can restore behavioral deficits in the Morris water maze task in the line 41 AD model.

### Aβ load is decreased by type I and type III NRG1

**Figure 2:**
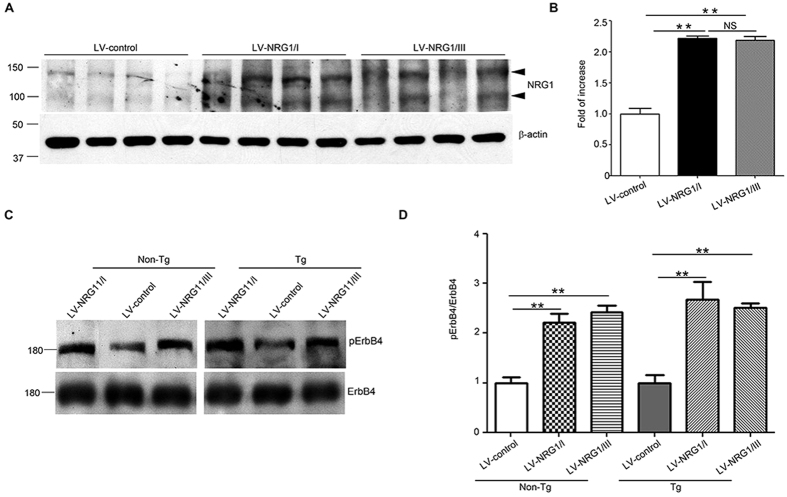
LV-NRG1 injection Increased expression of NRG1 protein and phosphorylation of ErbB4 in the hippocampus. (**A**) Western blotting analysis of protein extracts of the hippocampus of Tg mice injected with LV-control, LV-NRG1/I or LV-NRG1/III using antibody against NRG1. Arrowheads show the full-length NRG1 type III (~140 kDa) and NRG1 type I (~95 kDa) isoforms. β-actin was used for loading control. (**B**) Normalized levels of NRG1 show a marked increase by LV-NRG1/I and LV-NRG1/III. **p < 0.01; NS, there are no statistically significant differences between LV-NRG1/I and LV-NRG1/III groups. (**C**) Western blotting analysis of protein extracts of the hippocampus using antibody against p-ErbB4 and ErbB4. (**D**) Ratios of p-ErbB4/ErbB4 show a significant increase of phosphorylation by LV-NRG1/I and LV-NRG1/III. **p < 0.01.

**Figure 3:**
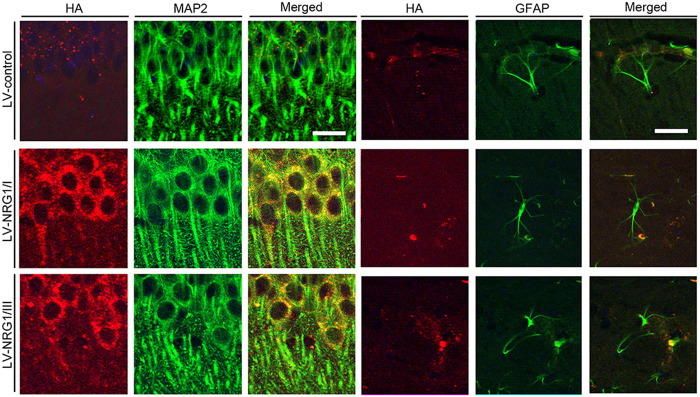
LV-NRG1/I or LV-NRG1/III primarily infected MAP2 + pyramidal neurons. Representative co-immunostaining for HA epitope with neuronal marker (MAP2) and astrocytic marker (GFAP) to label cells transduced by lentiviruses *in vivo*. Results show that LV-NRG1/I and LV-NRG1/III primarily infected MAP2^+^ pyramidal neurons with a small percentage of GFAP^+^ astrocytes. Although MAP2 is primarily dendritic marker, it can be detected in soma depending on levels of expression.

We determined whether expression of exogenous NRG1 is elevated following bilateral viral injection into the hippocampus of non-Tg and APP-Tg mice. Levels of endogenous NRG1 protein in the brain showed no differences between Non-Tg and TG (Supplemental Figure S1). Following viral injection, levels of NRG1 protein were 2-fold higher in mice injected with LV-NRG1/I or LV-NRG1/III LV than those in controls (Fig. 2A,B). Furthermore, levels of phosphorylation of ErbB4 in LV-NRG1 were 2.5 fold higher than those in LV-control (Fig. 2C,D), supporting activation of ErbB4-mediated signaling pathways. Immunostaining shows that the majority of cells being transduced are MAP2-positive pyramidal neurons, though a small percentage of GFAP–positive astrocytes were also transduced (Fig. 3).

**Figure 4:**
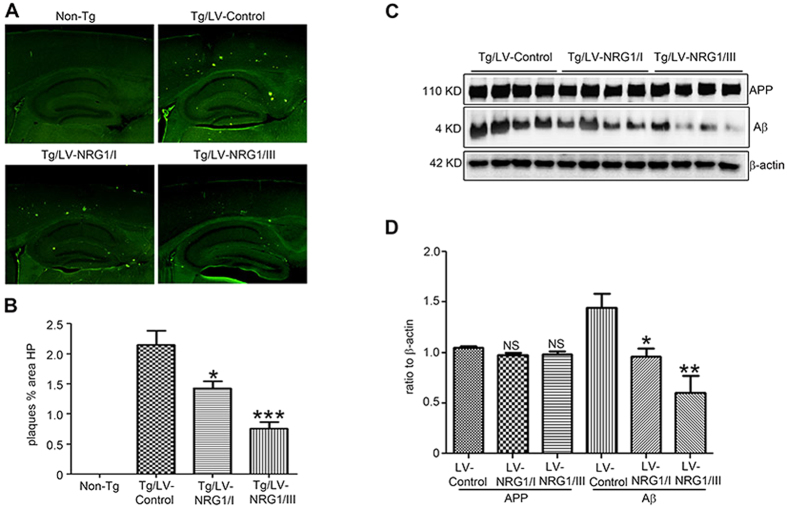
Aβ load is decreased by type I and type III NRG1. (**A**) Representative immunostaining for plaques on sections of the hippocampus of non-Tg and APP-Tg injected with LV-control, LV-NRG1/I or LV-NRG1/III. (**B**) The area of neuropil occupied by Aβ-immunoreactivity was calculated from 9 randomly selected sections per mouse (N = 3). (**C**) Western blotting analysis of protein extracts of the hippocampus using anti-Aβ antibodies 6E10. β-actin was used for protein loading control. (**D**) Normalized levels of Aβ show a marked decrease by LV-NRG1/I and LV-NRG1/III. The results were expressed as mean ± SEM. *p < 0.05; **p < 0.01;***p < 0.0005, NS, not significant.

To determine whether elevated levels of NRG1 affect Aβ load, brain sections were immunostained with antibodies specifically detecting human Aβ (Fig. 4A). To quantify amyloid deposition, the area of neuropil occupied by Aβ-immunoreactivity was calculated from 9 randomly selected sections per mouse. Type I and type III NRG1s decreased amyloid deposition by 37% and 66% compared to control viruses, respectively (Fig. 4B). Consistent with this finding, levels of Aβ were markedly reduced in the APP-Tg injected with LV-NRG1/I or LV-NRG1/III, though levels of APP were not altered (Fig. 4C,D). These results indicate that both type I and type III NRG1s can reduce Aβ load in the line 41 AD mouse model.

### Neuropathology is ameliorated by type I and type III NRG1

**Figure 5:**
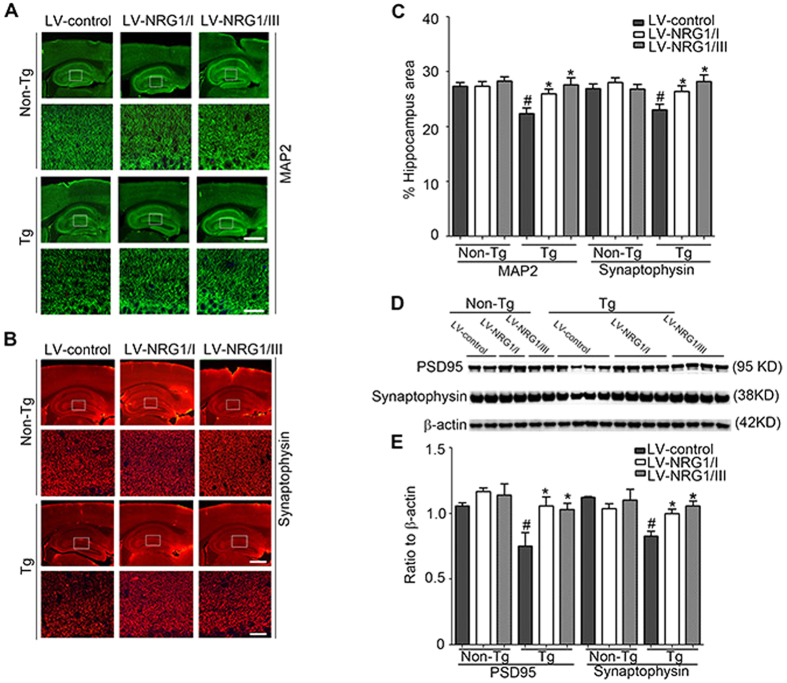
Neuropathology is ameliorated by type I and type III NRG1. Representative immunostaining for MAP2 (**A**) and presynaptic marker, synaptophysin (**B**) on sections of the hippocampus of non-Tg and APP- Tg injected with LV-control, LV-NRG1/I or LV-NRG1/III. The area of neuropil occupied by MAP2- or synaptophysin-immunoreactivity was calculated from 9 randomly selected sections per mouse (N = 3) (**C**). Although MAP2 is primarily a dendritic marker, it can be detected in soma depending on levels of expression. (**D**) Western blotting analysis of protein extracts of the hippocampus using antibodies against synaptophysin and the postsynaptic marker, PSD95. β-actin was used for a protein loading control. (**E**) Normalized levels of synaptophysin and PSD95 show a marked increase by LV-NRG1/I and LV-NRG1/III. The results were expressed as mean ± SEM. *p < 0.05. ^#^p < 0.01, denotes a statistically significant difference between Non-Tg/LV-control and Tg/LV-control for the respective markers in panels C and E.

Aβ neurotoxicity is known to affect multiple compartments of neurons, including dendrites and synapses. To determine whether type I or type III NRG1 reduces dendritic and synaptic alterations in APP-Tg mice, immunohistochemistry and Western blotting analysis were performed. As shown in Fig. 5A, MAP2 immunoreactivity is markedly reduced in APP-Tg mice as compared to that in non-Tg controls. The reduction is ameliorated by both type I and type III NRG1s (Fig. 5A). Similarly, a reduction in the presynaptic marker, synaptophysin, was observed in the APP- Tg mice (Fig. 5B,C). The alteration was reversed by both type I and type III NRG1 (Fig. 5B,C). Consistent with immunohistochemical analysis, Western blotting analysis confirmed that levels of synaptophysin in mice injected with type I or type III NRG1 were comparable to those in non-Tg controls (Fig. 5D,E). When the postsynaptic marker, PSD95, was analyzed, similar results were observed (Fig. 5E). These results demonstrated that neuropathology in AD mice was ameliorated by both type I and type III NRG1.

### Type I and type III NRG1 increase expression of Aβ-degrading enzyme Neprilysin

**Figure 6:**
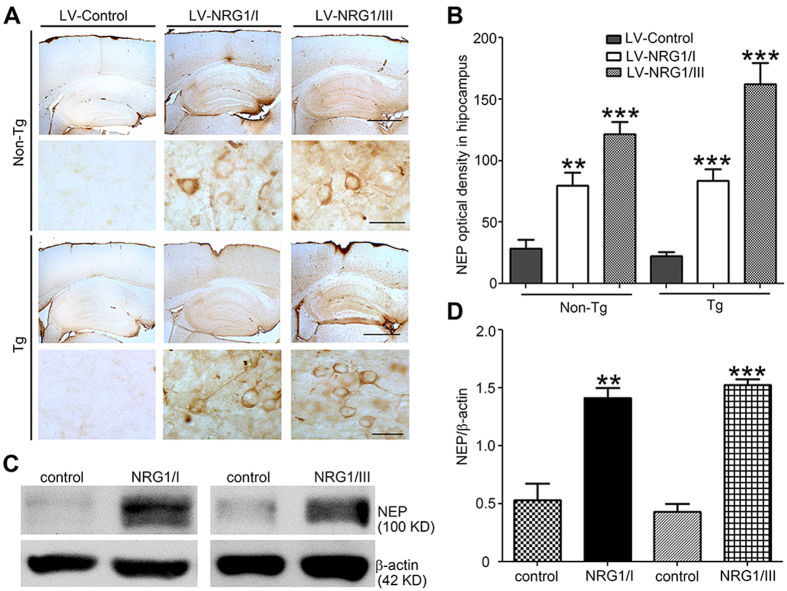
Type I and type III NRG1 increase expression of Neprilysin in mice and *in vitro*. Representative immunostaining for NEP (**A**) on sections of the hippocampus of non-Tg and APP-Tg injected with LV-control, LV-NRG1/I or LV-NRG1/III. (**B**) The area of neuropil occupied by NEP-immunoreactivity was calculated from 9 randomly selected sections per mouse (N = 3). (**C**) Western blotting analysis of NEP expression in primary hippocampal neuron cultures treated with 50 ng/ml recombinant soluble type I NRG1 and soluble type III NRG1, respectively. β-actin was used for loading control. Normalized levels of NEP show a marked increase of NEP by NRG1/I and NRG1/III. The results were expressed as mean ± SEM. **p < 0.01;***p < 0.0005.

Because Aβ load was decreased by both type I and type III NRG1, we next sought to examine potential mechanisms underlying the effects of NRG1 overexpression on amyloid plaque numbers. Several lines of evidence led us to examine whether NRG1, acting through ErbB receptors, induces expression or activity of the NEP. First, NEP is one of the most potent Aβ-degrading enzymes^27^. Second, NRG1 promotes muscle regeneration^28^, and NEP is also expressed and implicated in muscle regeneration^29^. Immunostaining shows that the immunoreactivity of NEP was dramatically increased in mouse brains injected with LV-NRG1/I or LV-NRG1/III (Fig. 6A,B). Next, we sought to study if NRG1 can regulate NEP expression *in vitro*. We demonstrated that levels of NEP were elevated in primary hippocampal neurons treated with 50 ng/ml soluble recombinant type I human NRG1 or type III NRG1 (Fig. 6C,D) for 48 hours. These results indicate that the soluble ectodomains of both type I and type III NRG1 are able to increase expression of NEP with comparable potency. To gain mechanistic insights into NRG1-regulated NEP expression, a luciferase reporter construct containing a 2.5 kb human NEP promoter^30^ was transfected into NB7 cells, which express endogenous NEP (data not shown). The results showed that both soluble type I and type III NRG1s significantly stimulate NEP promoter activity (Supplemental Figure S2). These results suggest that NRG1 increases NEP expression in part through transcriptional activation of the NEP promoter.

## Discussion

In the present study, we demonstrate that exogenous NRG1 improves deficits in Morris water maze behavioral task and neuropathology in a mouse line 41 model of AD. Aβ load is markedly reduced. Consistent with these results, solube ectodomains of both type I and type III NRG1s induce expression of Aβ-degradading enzyme NEP in primary hippocampal neuronal cultures. Our results suggest that NRG1 is a therapeutic target for the treatment of AD, but additional studies are needed before devising the strategy of using NRG1 itself or agents modulating NRG1-mediated signaling pathways for therapeutic purposes.

In addition to increasing NEP expression, NRG1 may improve deficits in AD mice via other mechanisms. NRG1 signaling has been shown to play a role in synaptic differentiation and function in the CNS. For example, NRG1 increases dendritic spine size^24^, modulates long-term potentiation at CA1 synapses^31^,^32^,^33^ and enhances entorhinal-hippocampal synaptic transmission^34^. NRG1 enhances depolarization-induced GABA release in the prefrontal cortex^35^. NRG1 also modulates hippocampal gamma oscillations^36^ that are thought to synchronize neuronal networks during learning and memory. Interestingly, recent results demonstrate that deficits in inhibitory interneurons link altered network activity and cognitive dysfunction in an AD model^37^,^38^. Therefore, further investigation is needed to determine if elevated expression of NRG1 modulates synaptic function in APP-Tg. Furthermore, ErbB4 is expressed in interneurons^39^, whereas ErbB2 and ErbB3 receptors are expressed in glial cells. Thus, NRG1 may affect neuronal structure and function by indirectly regulating the function of glia^40^.

While our results support beneficial effects of NRG1, transgenic overexpression of full length type I or type III NRG1 impairs cortical functions^41^,^42^. The discrepancy may be due to differences in approaches. The transgenic approach is different from lentivirus-mediated transduction utilized in our study in terms of the timing, brain regions and levels of NRG1 overexpression. For example, in Agarwal *et al*. expression levels of type III NRG1 were four-fold higher relative to controls in the whole brain starting from the E16 stage and onwards. Thus, the timing and the target cell populations of NRG1 overexpression are quite different from our paradigm of lentivirus transduction in the hippocampus of adult mice. Similarly, in Yin *et al*. NRG1 type I was selectively increased under the control of the CamK2α promoter in excitatory neurons in multiple forebrain regions, including the prefrontal cortex, the hippocampal and the striatum. Furthermore, overexpression of exogenous NRG1 starts at neonatal stages. Finally, when expressed at modest levels, NEP has been shown to afford beneficial effects in AD mice^8^,^43^,^44^,^45^. It is worth noting that much higher levels of NEP are implicated in side effects *in vivo* likely due to non-specific activity of NEP on other targets^46^.

The human mutant APP transgenic mouse is an imperfect AD model^47^. For example, it contains multiple copies of mutated human APP cDNA under the control of artificial promoters that may not recapitulate the dysregulated expression patterns *in vivo*. In addition to elevated levels of Aβ, the resulting high levels of APP and other processed APP fragments may cause unexpected effects^47^. To circumvent this problem, Saito *et al*. generated a single humanized APP mutation knock-in AD mouse model^47^. Hence, it warrants future testing of NRG1 treatment in other AD models, including the single humanized APP mutation knock-in AD mouse model^47^ and the triple transgenic mice that display both plaque and tangle pathology^48^. Furthermore, in contrast to the finding of a positive correlation of SNP rs392499 in U.S. AD patients^14^, there is no such a correlation in U.K. AD patients^49^. Middle *et al*. suggest that the discrepancy may be due to several differences in both studies, including the design on selection of cohorts and haplotype analysis programs. As there are additional SNPs, an in-depth analysis of other variants needs to be performed with the same experimental design.

Finally, as peripheral delivery of soluble NRG1 has been shown to be capable of crossing the blood brain barrier (BBB)^50^,^51^, the results raise the possibility of devising a strategy for an enhanced ability to cross the BBB^52^ and for a regulated delivery of NRG1 from a peripheral tissue such as muscle. Taken together, these and our studies add valuable information in considering how to control spatiotemporal regulation and expression levels of exogenous NRG1 as a treatment for AD.

## Methods

### Antibodies

Antibodies include monoclonal antibody against NEP (clone Ab951) and PSD95 (Abcam), rabbit polyclonal antibody against NRG1 (clone SC-348) and anti-ErbB4 (Santa Cruz), anti-phospho-ErbB4 (Tyr1284) (Cell Signaling), mouse anti-β-actin antibody (Sigma), mouse monoclonal Aβ antibody 6E10 (Covance) and synaptophysin (Millipore).

### Recombinant soluble Neuregulin 1 and NRG1-expressing lentiviral vectors

Recombinant soluble human type I NRG1 (NDFβ1_14-246_) was obtained from Amgen (Thousand Oaks, CA). Recombinant human type III NRG1 (SMDF, catalogue # 378-SM-025) was purchased from R&D systems (Minneapolis, MN). Lentiviruses expressing rat β1, a full length type I or type III NRG1^53^ under the CMV promoter, were generated using the ViraPower Lentiviral Expression System (Invitrogen) and were produced by the Gene Transfer Core at the Salk Institute.

### Animals and Viral injection

Transgenic mice line 41 expressing hAPP751 cDNA containing the London (V717I) and Swedish (K670M/N671L) mutations under the regulatory control of the murine Thy-1 gene was reported previously^25^. The increased Aβ plaque burden was found in the cortex and hippocampus starting at three to four months in this line 41 transgenic mouse^25^. These mice already show significant learning and memory deficits in the Morris water maze by six months of age^54^. To investigate if exogenous NRG1 can improve cognitive impairment and ameliorate neuropathology post-symptomatically, 7- month old APP-Tg and age-matched control littermates were divided into 6 groups (N = 6) and received bilateral injections of LV-control, LV-NRG1/I or LV-NRG1/III, respectively. To avoid any compounding factors associated with sex, only female non-Tg and APP-Tg mice were included. Stereotaxic injection was performed using a Hamilton syringe connected to a hydraulic system at 1 μl every 2 min. A total 2 μl of lentivirus was infused into each hippocampus. The needle was left in place for 5 min after completion of injection. Coordinates for viral injection of the hippocampus are (AP-2, ML ± 1.5, and DV-1.3). All experiments described were carried out in strict accordance with good animal practice according to NIH recommendations. All procedures for animal use were approved by the Institutional Animal Care and Use Committee at the Salk Institute.

### Water maze test

The water maze test was performed as previously described^26^,^44^. For this purpose, a pool (diameter 180 cm) was filled with opaque water (24 °C) and mice were first trained to locate a visible platform (days 1–3) and then a submerged hidden platform (days 4–7) in four daily trials 2–3 min apart. Mice that failed to find the hidden platform within 90 seconds were placed on it for 30 seconds. The same platform location was used for all sessions and all mice. The starting point at which each mouse was placed in the water was changed randomly between two alternative entry points located at a similar distance from the platform. Time to reach the platform (latency), path length, and swim speed were recorded with a Noldus Instruments EthoVision video tracking system (San Diego Instruments) set to analyze two samples per second. The data were calculated by a method previously described^26^. The means of latency or distance across all four trials daily from day 1 to day 3 for visible platform and from day 4 to day 7 for hidden platform were calcuated. Repeated measures ANOVA followed by Fisher’s Least Significant Difference post-hoc test was used in the analyses with level of significance set at p < 0.05. A two-tailed Student t-test was utilized where appropriate, with calculated comparisons of p < 0.05 considered significant. All reported values represent the means ± standard error of the mean (SEM).

### Analysis of NEP expression and neurodegeneration

To verify the expression levels of NEP, vibratome sections were immunolabeled with a monoclonal antibody against NEP (CD10, Abcam) and detected with the Tyramide Signal AmplificationTM-Direct (Red) system (NEN Life Sciences). All sections were processed simultaneously under the same conditions, and immunostaining procedures were performed twice to assess reproducibility. Sections were imaged with a Zeiss 63X (N.A. 1.4) objective on an Axiovert 35 microscope (Zeiss) with an attached MRC1024 LSCM system (BioRad). To confirm the specificity of primary antibodies, control experiments were performed where sections were incubated overnight in the absence of primary antibody (deleted) or preimmune serum and primary antibody alone. The integrity of the neuronal structure was evaluated as previously described^54^,^55^; briefly, blind-coded, 40 μm thick vibratome sections from mouse brains fixed in 4% paraformaldehyde were immunolabeled with the mouse monoclonal antibodies against MAP2 (dendritic marker), synaptophysin (presynaptic marker, Millipore) and PSD95 (postsynaptic marker, Abcam). After overnight incubation, sections were incubated with the Tyramide Signal Amplification TM-Direct (Red) system (NEN Life Sciences), transferred to SuperFrost slides (Fisher Scientific) and mounted under glass coverslips with anti-fading media (Vector Laboratories). All sections were processed under the same standardized conditions. The immunolabeled blind-coded sections were serially imaged with the LSCM (MRC1024, BioRad) and analyzed with the ImageJ 1.43 program (NIH), as previously described^8^. For each mouse, a total of 3 sections were analyzed and for each section, 4 fields in the frontal cortex and hippocampus were examined. Results were expressed as percent area of the neuropil occupied.

### Analysis of Aβ plaque load

Amyloid plaque load was quantified as previously described^8^. Vibratome sections were incubated overnight with mouse monoclonal antibody 6E10 (1:600), which specifically recognizes human Aβ, followed by FITC-conjugated anti-mouse IgG. The FITC-labeled sections were imaged with the LSCM as described previously^8^. Digitized images were analyzed with NIH Image 1.43 to determine % area of neuropil occupied by Aβ immunoreactive deposits in hippocampus. Three immunolabeled sections were analyzed per mouse.

### Determination of APP levels, APP products and Aβ-degrading enzymes

Levels of APP immunoreactivity were determined in brain homogenates by WB and in vibratome sections by ICC, as previously described^25^.

### Hippocampal neuron cultures

Hippocampi from E18 mouse embryos were dissected free of meninges, minced, washed in HBSS and digested with 0.25% trypsin (Worthington) plus 0.05 mg/ml DNase I (Worthington) for 15 minutes at 37° C. Trypsinization was stopped with DMEM supplemented with 10% FBS and tissue was washed twice and subsequently dissociated by triturating 10 times with a flamed Pasteur pipet. Cells were passed through a 70 μm strainer (Corning) and plated at a density of 7 × 10^5^ cells/well in poly-L-lysine (Sigma) coated 6-well plates in DMEM supplemented with 10% FBS. Four hours later, medium was removed and replaced with Neurobasal medium supplemented with 2% B27 (Invitrogen) and 100 units/ml penicillin and 100 μg/ml streptomycin. Subsequently, half volume of Neurobasal medium in each well was replaced twice a week. After one week, hippocampal neurons were treated with 50 ng/ml recombinant soluble type I or type III for 48 hours, respectively. For all treatments, cells were treated in triplicates and experiments were repeated a minimum of three times to ensure reproducibility.

## Additional Information

**How to cite this article**: Xu, J. *et al*. Neuregulin 1 improves cognitive deficits and neuropathology in an Alzheimer's disease model. *Sci. Rep.* **6**, 31692; doi: 10.1038/srep31692 (2016).

## Supplementary Material

Supplementary Information
