# Author Correction: Neuregulin 1 improves cognitive deficits and neuropathology in an Alzheimer’s disease model

**DOI:** 10.1038/s41598-024-83259-w

**Published:** 2025-01-21

**Authors:** Jiqing Xu, Fred de Winter, Catherine Farrokhi, Edward Rockenstein, Michael Mante, Anthony Adame, Jonathan Cook, Xin Jin, Eliezer Masliah, Kuo-Fen Lee

**Affiliations:** 1 Clayton Foundation for Peptide Biology Laboratories, The Salk Institute, La Jolla, CA 92037 USA; 2 Department of Neurosciences, University of California at San Diego, La Jolla, CA 92093 USA; 3 Molecular Neurobiology Laboratories, The Salk Institute, La Jolla, CA 92037 USA

Correction to: *Scientific Reports* 10.1038/srep31692, published online 25 August 2016

This Article contains an error in Figure 5.

As a result of an error during figure assembly, images originating from the same sample were used in Figure 5A LV-control/Non-Tg and LV-NRG1/I/Non-Tg resulting in partial overlap. The correct Figure 5A and accompanying legend appear below.

**Fig. 5:**
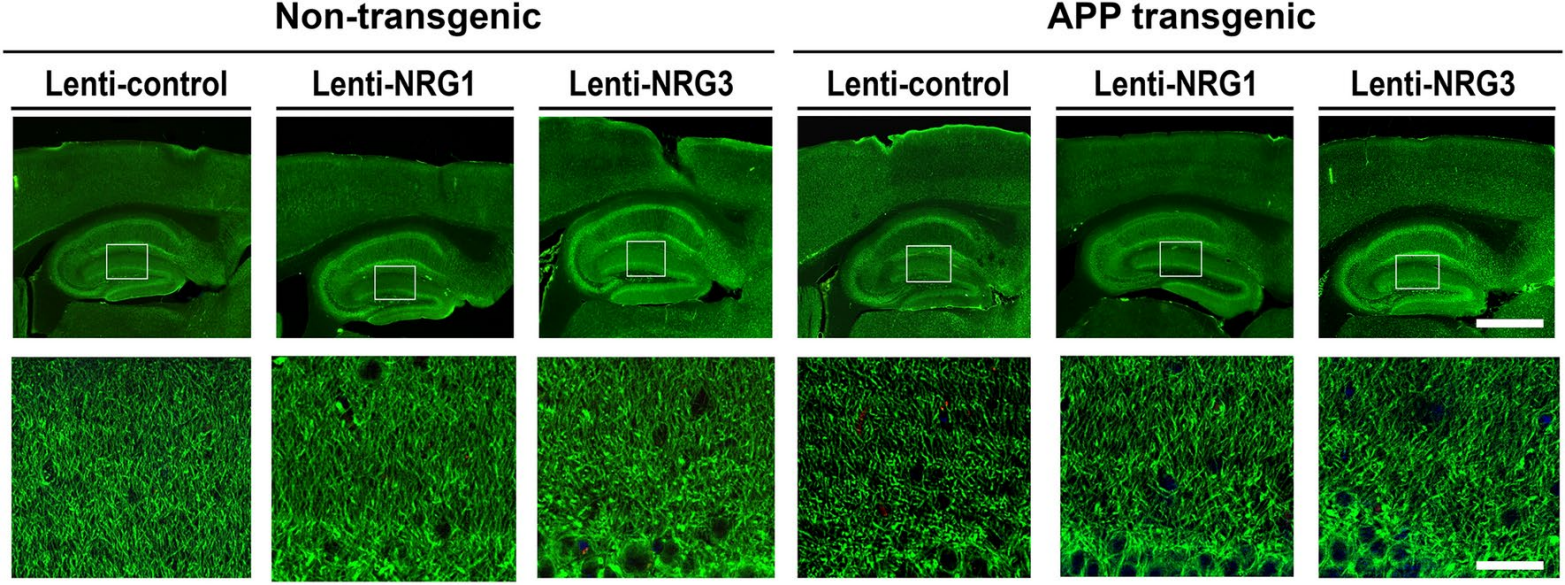
Representative immunostaining for MAP2 (**A**) and presynaptic marker, synaptophysin (**B**) on sections of the hippocampus of non-Tg and APP-Tg injected with LV-control, LV-NRG1/I or LV-NRG1/III. The area of neuropil occupied by MAP2- or synaptophysin-immunoreactivity was calculated from 9 randomly selected sections per mouse (N = 3) (**C**). Although MAP2 is primarily a dendritic marker, it can be detected in soma depending on levels of expression. (**D**) Western blotting analysis of protein extracts of the hippocampus using antibodies against synaptophysin and the postsynaptic marker, PSD95. β-actin was used for a protein loading control. (**E**) Normalized levels of synaptophysin and PSD95 show a marked increase by LV-NRG1/I and LV-NRG1/III. The results were expressed as mean ± SEM. *p < 0.05. ^#^p < 0.01, denotes a statistically significant difference between Non-Tg/LV-control and Tg/LV-control for the respective markers in (**C,E**).

