# Supplementary material for: Neuregulin 1 improves cognitive deficits and neuropathology in an Alzheimer’s disease model
Source: Sci Rep. 2016 Aug 25;6:31692. doi: 10.1038/srep31692 (PMC4997345; doi:10.1038/srep31692)
Supplement: Supplementary Information [file srep31692-s1.pdf]

Supplementary information

Neuregulin 1 improves cognitive deficits and neuropathology in an Alzheimer's disease model

Jiqing Xu[1], Fred DeWinter[1], Catherine Farrokhi[1], Edward Rockenstein[2], Michael Mante[2], Anthony Adame[2], Jonathan Cook[3], Xin Jin[3], Eliezer Masliah[2] and Kuo-Fen Lee[1*]

[1]Clayton Foundation for Peptide Biology Laboratories, The Salk Institute, La Jolla, CA 92037
[2]Department of Neurosciences, University of California at San Diego, La Jolla, CA 92093
[3]Molecular Neurobiology Laboratories, The Salk Institute, La Jolla, CA92037

*Corresponding Author:
Kuo-Fen Lee
Clayton Foundation for Peptide Biology Laboratories, The Salk Institute, La Jolla, CA 92037
E-mail: klee@salk.edu

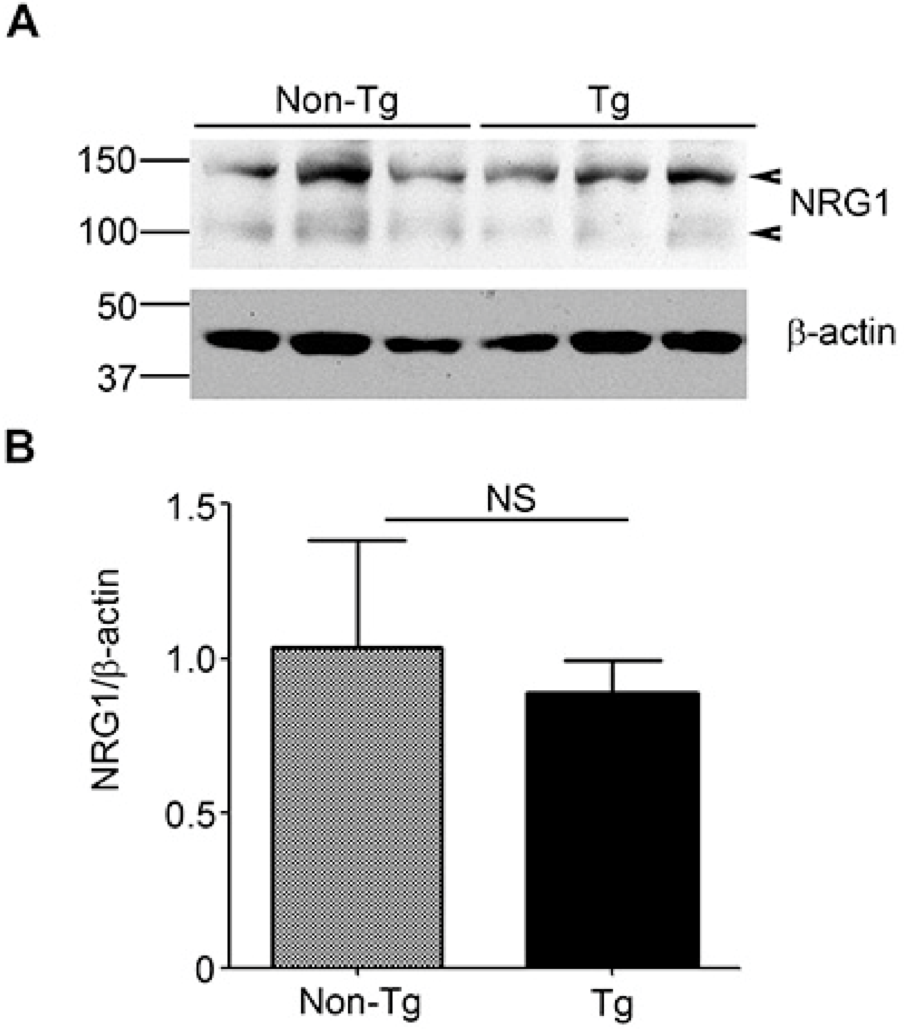

Supplemental Figure S1. No significant differences in expression of NRG1 in the brain between Non-Tg and Tg mice

(A) Western blotting analysis of NRG1 protein in the brain of 7-month old Non-Tg and Tg mice. β-actin was used for protein loading control. (B) No significant (NS) differences in levels of NRG1 were found.

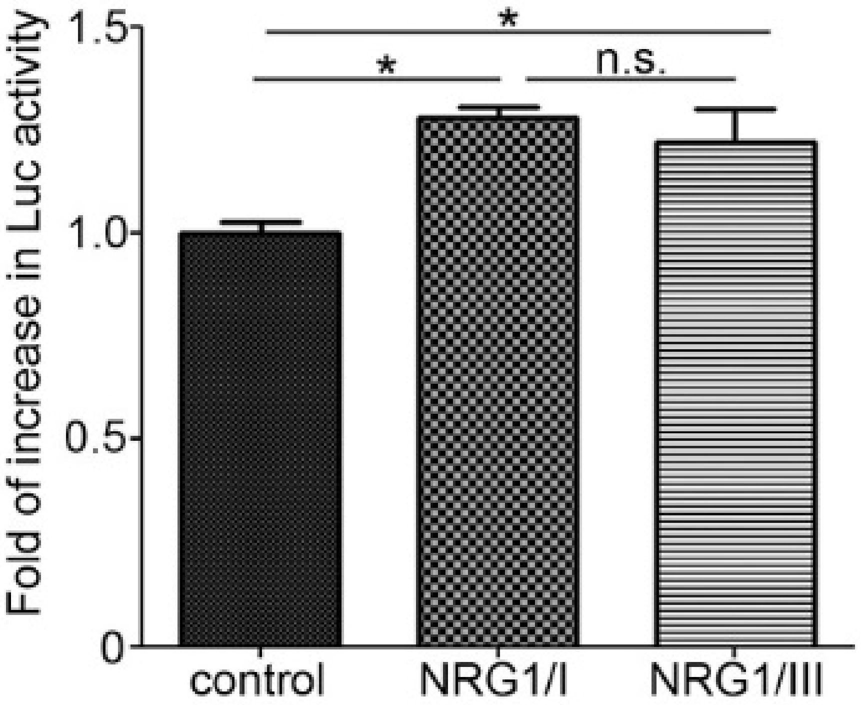

Supplemental Figure S2. Soluble NRG1 stimulates the human neprilysin promoter in neuroblastoma cells

NB7 cells were co-transfected with the luciferase vector containing the 2.5 kb human NEP promoter and pRSV-βgal for 24 hours and treated with 50 ng/ml recombinant soluble type I NRG1 and type III NRG1s, respectively. Cells were lysed 24 hours later and subjected to luciferase activity and β–gal assays. Normalized data shows that both soluble type I and type III NRG1s stimulate NEP promoter activity. $p<0.05$; NS, not significant.
